# Astragalus Flavone Ameliorates Atherosclerosis and Hepatic Steatosis Via Inhibiting Lipid-Disorder and Inflammation in apoE^−/−^ Mice

**DOI:** 10.3389/fphar.2020.610550

**Published:** 2020-12-14

**Authors:** Chuanrui Ma, Jing Zhang, Shu Yang, Yunqing Hua, Jing Su, Yuna Shang, Zhongyan Wang, Ke Feng, Jian Zhang, Xiaoxiao Yang, Hao Zhang, Jingyuan Mao, Guanwei Fan

**Affiliations:** ^1^ First Teaching Hospital of Tianjin University of Traditional Chinese Medicine, Tianjin, China; ^2^ Tianjin Key Laboratory of Translational Research of TCM Prescription and Syndrome, Tianjin, China; ^3^ Department of Endocrinology, The Second Clinical Medical College, Shenzhen People’s Hospital, Jinan University, Shenzhen, China; ^4^ Integrated Chinese and Western Medicine Postdoctoral Research Station, Jinan University, Guangzhou, China; ^5^ Tianjin State Key Laboratory of Component-Based Chinese Medicine, Tianjin University of Traditional Chinese Medicine, Tianjin, China; ^6^ College of Life Sciences, Nankai University, Tianjin, China; ^7^ Tianjin Key Laboratory of Radiation Medicine and Molecular Nuclear Medicine, Institute of Radiation Medicine, Chinese Academy of Medical Sciences and Peking Union Medical College, Tianjin, China; ^8^ Department of Pharmacology, College of Basic Medical Sciences, Tianjin Medical University, Tianjin, China; ^9^ Key Laboratory of Metabolism and Regulation for Major Diseases of Anhui Higher Education Institutes, College of Food and Biological Engineering, Hefei University of Technology, Hefei, China

**Keywords:** Astragalus flavone, inflammation, atherosclerosis, hepatic steatosis, lipid disorder

## Abstract

Atherosclerosis is a major pathogenic driver of cardiovascular diseases. Foam cell formation plays a key role in atherogenesis, which is affected by lipid disorder and inflammation. Therefore, inhibition of foam cell formation is a therapeutic approach for atherosclerosis treatment. Total flavone of *Astragalus membranaceus* (TFA) is extracted from *A. membranaceus* that has protective effect on cardiovascular disease. However, the effect of TFA on atherosclerosis and the underlying mechanism remains unknown. In this study, we determined whether TFA could inhibit atherosclerosis and uncovered the underlying mechanism. *In vivo*, ApoE deficient mice were treated with TFA and high-fat diet for 16 weeks. Subsequently, atherosclerotic lesions, hepatic steatosis and associated genes expression *in vitro* and *in vivo* were determined. We found that TFA reduced atherosclerotic lesion size and enhanced plaque stability, which might be attributed to improved lipid disorder, reduced inflammation and decreased monocyte adhesion. Mechanistically, TFA inhibited hepatic steatosis via regulating the genes responsible for lipid metabolism, by which ameliorating the lipid disorder. Moreover, in macrophage, TFA reduced the expression of scavenger receptors such as CD36 and SRA; and promoted the expression of ATP-binding cassette transporter A1 and G1 (ABCA1/G1). More importantly, TFA reduced miR-33 expression and dampened NFκB activity, by which de-repressing ABCA1/G1 activity and inhibiting the inflammation. Collectively, TFA can attenuate atherosclerosis via dual suppression of miR-33 and NFκB pathway, and partially through inhibition of scavenger receptors in macrophage. In addition, TFA ameliorates the hepatic steatosis and lipid disorder, which in turn contributes to the amelioration of atherosclerosis, suggesting that TFA might be a novel therapeutic approach for inhibition of atherosclerosis and hepatic steatosis.

**GRAPHICAL ABSTRACT:**
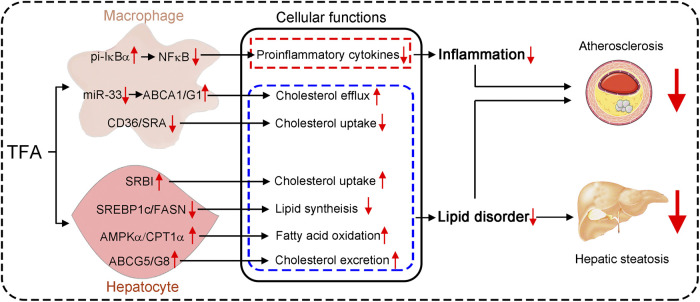
Astragalus flavone with different molecular weights have different effects on inflammation and lipid metabolism.

## Introduction

Atherosclerosis is the principal risk factor of cardiovascular diseases, which is mainly driven by lipid disorder and inflammation. Although the introduction of lipid-lowering therapies, such as 3-hydroxy-3-methylglutaryl coenzyme A reductase (HMGCR) inhibitors and proprotein convertase subtilisin-kexin type 9 (PCSK9) inhibitors have lowered the risk of cardiovascular disease. The atherosclerosis-driven cardiovascular disease remains the major causes of morbidity and mortality worldwide (de Winther and Lutgens, 2019). Thereby, new strategies to lower the risk of cardiovascular disease are still in need.

Lipid-enriched foam cells are the hallmark of atherosclerotic plaques (Moore and Tabas, 2011). Lipid accumulation in the cytoplasm can induce macrophages to transform into detrimental foam cells, thereby accelerating atherosclerotic plaque destabilization, rupture, and even thrombogenesis (Yang et al., 2018). Therefore, inhibition of foam cell formation is a key target for retarding atherogenic progression. High cholesterol uptake and low efflux in macrophage can lead to cellular lipid accumulation and the following foam cell formation (Yu et al., 2013), which is mainly determined by the transporters responsible for cholesterol efflux, such as ABCA1 and ABCG1 (Tall et al., 2008); and scavenger receptors in charge of cholesterol uptake, such as, SRA and CD36 (Crucet et al., 2013; de Winther et al., 2000; Nakata et al., 1999). Therefore, inhibition of cholesterol uptake or promotion of efflux is a promising antiatherogenic strategy (Tall, 2018; Ouimet et al., 2019). Liver is a critical organ that regulates lipid metabolism, which mainly determines the cholesterol metabolism and lipid profile in serum, thereby affecting the atherogenesis (Lee et al., 2018). Importantly, ABCA1/G1-mediated cholesterol efflux is the initial step of reverse cholesterol transport (RCT), by which cholesterol moves out of foam cells in atherosclerotic plaques into liver and finally into the feces.

Atherosclerosis is driven not only by dyslipidemia but also by inflammation. Chronic inflammation can coordinate with lipid dysfunction. More specifically, hyperlipidemia can skew plaque macrophages toward an atherogenic M1 phenotype instead of toward the antiatherogenic M2 phenotype (Barrett et al., 2019). Importantly, NF-κB is a critical signaling regulator that controls inflammation. In the unstimulated state, the IκB binds to NF-κB dimers in the cytoplasm to render NF-κB inactive. However, upon stimulation, IκB proteins are phosphorylated and subsequently degraded, which leads to release of active NF-κB dimers for nuclear translocation and target gene induction (Baeuerle and Baltimore, 1988). In addition, miRNAs can act as fine-tuners of genes expression, including genes responsible for lipid metabolism and inflammation (Feinberg and Moore, 2016). Among these miRNAs, miR-33 can regulate macrophage polarizaiton and inflammation (Ouimet et al., 2015). Moreover, miR-33 can limit cholesterol efflux capacity by restricting ABCA1/G1 activity in foam cells (Price et al., 2019). In contrast, inhibition of miR-33 can enhance RCT, raise plasma HDL, and lower VLDL (Rayner et al., 2010; Horie et al., 2012; Rayner et al., 2011a; Rayner et al., 2011b). Therefore, inhibition of miR-33 and NF-κB holds therapeutic promise for atherosclerosis treatment.

Flavone has shown the potential to improve HDL function through the effects on antioxidant status and anti-inflammation (Millar et al., 2017). Noticeable, the current study showed that higher intake of flavone was associated with a lower risk of coronary heart disease (Ma L. et al., 2020). Intriguingly, studies have shown that flavone can exert protective effect by modulating miRNAs expression in different model of diseases (Lam et al., 2012; Sun et al., 2015; Yin et al., 2015; Huang et al., 2016; Rigalli et al., 2016). Total flavone of *Astragalus membranaceus* (TFA) is extracted from *A. membranaceus* which is a traditional Chinese medicine that has protective effects on cardiovascular disease (Su et al., 2009; Liu et al., 2016). Moreover, TFA is easily available in technology and low-cost in economics. Therefore, in this study, we determined whether TFA could attenuate atherosclerosis and further uncovered the underlying mechanism. Given the important role of miR-33 and NF-κB pathway in cholesterol metabolism and inflammation, we postulated that TFA may inhibit atherosclerosis through regulating miR-33 expression and NF-κB pathway. In the current study, TFA reduced atherosclerosis and enhanced plaque stability in apoE deficient mice, which may be attributed to the improvement of lipid metabolism and inhibition of inflammation in liver and macrophage. Moreover, TFA suppressed the adhesion of monocytes to inflammatory endothelial cells. Mechanistically, these effects are associated with inactivation of miR-33 expression and NFκB activity as well as orchestrating genes responsible for lipid metabolism. Collectively, TFA may be a possible therapeutic intervention for atherosclerosis.

## Materials and Methods

### Reagents

Mouse anti-ICAM-1(Cat#: sc107), VCAM-1(Cat#: sc13160), GAPDH (Cat#: sc365062) SRA (Cat#: sc56777), αSMA (Cat#: sc130617), CD36 (Cat#: sc70644), and CD68 (Cat#: sc20060) monoclonal antibodies were purchased from Santa Cruz Biotechnology, Inc (Santa Cruz, CA). Rabbit anti-ABCG1 (Cat#: NB400-132), ABCA1(Cat#: NB400-105) and SRBI (Cat#: NB400-104) polyclonal antibodies were purchased from Novus Biologicals (Littleton, CO). Mouse anti-IL-1β (Cat#: #12242) monoclonal antibody was purchased from Cell Signaling Technology. Mouse anti-Arg (Cat#: ab239731) monoclonal antibody were purchased from Abcam (Cambridge, MA). Mouse anti-rabbit IgG-R (Cat#: sc2492), mouse anti-rabbit IgG-FITC (Cat#: sc2359) and m-IgGκ BP-FITC (Cat#: sc516140) antibodies were purchased from Santa Cruz Biotechnology, Inc (Santa Cruz, CA). Astragalus flavone (Cat#: SA9780) was purchased from Solarbio (Beijing, China).

### Cell Culture

Human umbilical vein endothelial cells (HUVECs) were cultured in VascuLife basal medium containing VEGF lifeFactors Kit (Lifeline Cell Technology, Frederick, MD). RAW264.7, THP-1 cells and peritoneal macrophages were cultured in complete RPMI1640 medium containing 10% FBS, 50 μg mL^−1^ penicillin/streptomycin and 2 mM glutamine.

### miR-33 Mimic Transfection in Macrophages

Macrophages were seeded into plate and cultured to 60% confluence. Cells were then transfected with 50 nM miR-33 mimic or miR-33 control (Guangzhou RiboBio Co., Ltd.) using Lipofectamine RNAiMAX (Invitrogen, Grand Island, NE) according to the manufacturer’s instructions. The medium was replaced with fresh medium after 6 h transfection.

### Animals and Treatment Schedule

The protocol for *in vivo* studies was approved by the Ethics Committee of Tianjin University of Traditional Chinese Medicine and conforms to the Guide for the Care and Use of Laboratory Animals published by the NIH (NIH publication, eighth edition, updated 2011). Eight weeks old, male ApoE^−/−^ mice were purchased from Beijing Vital River Laboratory Animal Technology Co., Ltd. The animals were housed in SPF units of the Animal Center at Tianjin University of Traditional Chinese Medicine in the environment with 60–70% humidity at temperature 22 ± 1 C and 12 h light-dark cycle. The mice can freely access to water and high-fat diet (41% fat plus 0.5% cholesterol, MD12015A, Medicience Ltd., China). The ApoE^−/−^ mice were randomly divided into three groups and fed HFD, HFD containing high dose TFA [low dose TFA-L, 10 mg day^−1^ kg^−1^ bodyweight (mpk)], high dose TFA (TFA-L, 20 mpk) for 16 weeks. The mice were daily checked for food intake, water drink and bodyweight gain during the treatment. At the end of experiment, all mice were anesthetized and euthanized as we previously reported (Ma C. et al., 2020; Ma et al., 2018), following by collection of aortas, peritoneal macrophages, blood samples and other tissues. Serum was prepared to determine lipid profile, including total cholesterol (Total-C, TC), high-density lipoprotein (HDL)-C, low-density lipoprotein (LDL)-C, and triglycerides (TG) (Ma et al., 2018). Mouse ox-LDL level was evaluated by the commercially provided ELISA kit.

### Atherosclerotic Lesion Analysis

The aortas were collected and used to prepare aortic root cross sections followed by determination of *en face* and sinus lesions with Oil Red O staining (Ma et al., 2018). All the images were obtained with a microscope and quantified lesion areas in *en face* aorta and aortic root cross sections, respectively. The lesion areas were expressed as μm^2^ or % of the total surface area. Necrotic core, fibrous cap, cellular apoptosis, collagen content, and expression of CD68, αSMA, Arg1, and IL-1β in lesion within aortic root cross sections were determined by Haematoxylin and eosin (H&E), sirius red staining, TUNEL staining, and immunofluorescent staining, respectively (Ma et al., 2018). The vulnerability index of plaques was calculated as (macrophage staining% + lipid staining%)/(SMCs% + collagen fiber%), according to a previous report (Williams et al., 2002).

### Hepatic Steatosis Analysis

The hepatic steatosis was evaluated as previously described (Ma et al., 2018; Ma C. et al., 2020; Wang et al., 2020). Briefly, after sacrifice, the liver was isolated and photographed to exhibit the color and size. In addition, 5μm frozen section of liver was prepared to perform HE and Oil red O staining. Furthermore, total cholesterol and triglyceride was detected using commercially provided kit.

### Determination of Foam Cell Formation *in vitro* and *in vivo*

Foam cell formation *in vitro* and *in vivo* was determined by Oil Red O staining as we previously described (Ma et al., 2018; Ma C. et al., 2020). Briefly, *in vivo*, peritoneal macrophages were isolated from TFA-treated apoE^−/−^ mice and seeded on cover slips in 24-well plates, and then stained with Oil Red O solution. *In vitro*, RAW264.7 cells were seeded on cover slips in 24-well plates. Cells were incubated with oxLDL for 3 h following by TFA treatment for 16 h. Cells containing lipid droplets (>10/cell) were considered as foam cells, and >10 fields/sample were counted.

### Cholesterol Uptake and Efflux Assay

Macrophages were incubated in medium containing DiI-oxLDL or 3-dodecanoyl-NBD cholesterol to evaluate ability of cholesterol uptake and efflux as previously studied (Ma C. et al., 2020). Briefly, in 12-well plates, macrophages (1.0 × 10^6^ cells/well) pretreated with TFA at different doses were incubated with 10 μg/mL Dil-oxLDL (Introvegen) in RPMI 1,640 at 37°C for 6 h. Fluorescence intensity was examined by a fluorescence microscopy.

The ability of macrophage cholesterol efflux was determined as we previous study (Ma C. et al., 2020). In brief, macrophage was firstly incubated with 3-dodecanoyl-NBD cholesterol (1 μg/mL, Cayman Chemical) for 6 h. And then cells were switched into serum-free medium containing both apo-AI (5 μg/mL) and HDL (20 μg/mL) as cholesterol acceptor in the presence or absence of TFA for 5 h. The fluorescence-tagged cholesterol in the medium and cells lysate was determined by the automatic microplate reader (Thermo Scientific, Varioskan Lux, USA). Cholesterol efflux was expressed as a ratio of fluorescence in the medium to the total amount of fluorescence in cells together with medium.

### Western Blot and Quantitative Real-Time PCR

Total cellular proteins were extracted from cells or liver tissue. Protein expression of ABCA1, ABCG1, CD36, GAPDH, FASN, SREBP1c, SRBI, IκBα, pi-IκBα, p65, pi-p65, and SRA were determined by Western blot (Yang et al., 2020).

Total RNA was isolated and purified, and cDNA was synthesized from 1 μg of total RNA using a reverse transcription kit (Vazyme bioteck co., ltd.). For quantitative real-time PCR (q-RT-PCR), specific genes were amplified by 40 cycles using SYBR green PCR master mix (DBI, Bioscience). Gene-specific primers are listed in Table S1. Expression of mRNA was normalized to the housekeeping gene GAPDH. miR-33 was normalized by U6 snRNA expression.

### Monocyte Adhesion Assay

The HUVECs were seeded and incubated in 24-well dishes. After reaching 85% confluence, HUVECs were preincubated with oxLDL (100 μg/ml), or a combination of different dose of TFA (6, 12, 24 μg/ml) with oxLDL (100 μg/ml) for 24 h. The THP-1 cells were then added to the 24-well dishes and incubated with HUVECs for 1h. Subsequently, unbound monocytes were removed by 3 times of washes with warm PBS. After washout, the adherent THP-1 cells to HUVECs were captured with a microscope and counted with ImageJ.

### Statistical Analysis

The data and statistical analysis complys with the recommendations on experimental design and analysis in pharmacology (Curtis et al., 2015). Data was expressed as means ± SEM and analyzed by using Graph Pad Prism software. One-way ANOVA for comparisons between multiple groups followed by Turkey’s method. The value of *p* ＜ 0.05 was considered statistically significant.

## Results

### TFA Attenuates Atherosclerotic Development in apoE^−/−^ Mice

**FIGURE 1:**
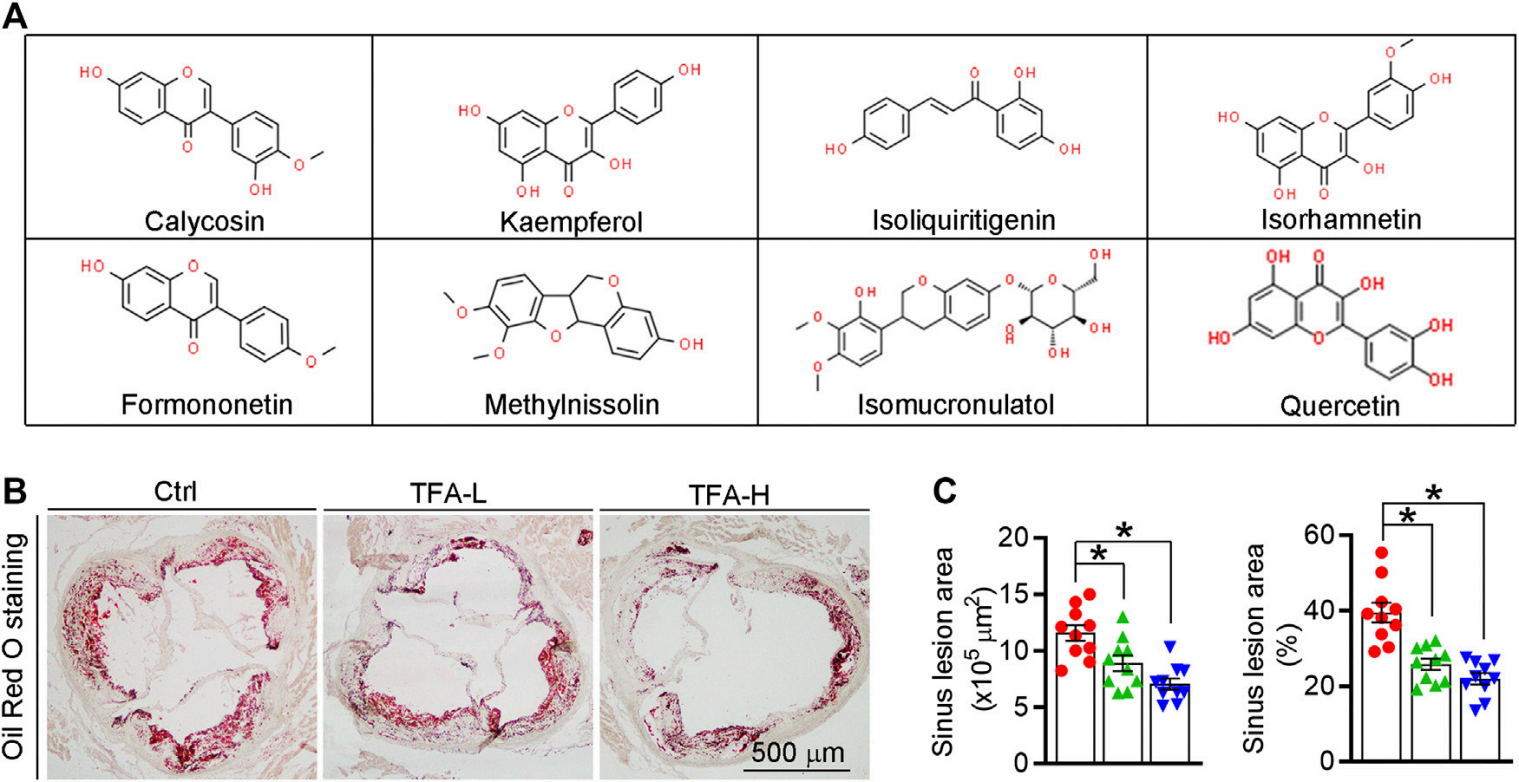
TFA reduces atherosclerotic lesion size in apoE^−/−^ mice. **(A)** The main ingredient of TFA. **(B, C)** Lesions in aortic root cross sections were determined by Oil Red O staining **(B)**, and quantified **(C)**. Lesion areas were expressed as μm^2^ and the ratio of lesion area to total area of aortic root cross sections, *n* = 10. Data are presented as mean ± SEM, *p < 0.05, significantly different from control; ns: not significantly different. TFA enhances atherosclerotic plaque stability by changing the plaque composition in apoE^−/−^ mice.

Total flavone of *A. membranaceus* (TFA) is extracted from *A. membranaceus* and the main ingredient of TFA includes calycosin, kaempferol, isoliquiritigenin, siorhamnetin, formononetin, methylnissolin, isomucronulatol, and quercetin(Lin et al., 2000; Zhao et al., 2008; Li et al., 2019) (Figure 1A). To test the hypothesis that long-term administration with TFA would protect against atherosclerosis, we treated atherogenic apoE^−/−^ mice with TFA and HFD for 16 weeks. After treatment, we evaluated atherosclerotic lesions. Analysis of atherosclerosis was assessed by Oil red O staining, which was followed by quantification as lesion area and the ratio of lesion area to total area of aorta or aortic root cross sections. Compared with mice that fed HFD alone, *en face* aortic lesions were markedly inhibited by TFA (Supplementary Figures S1A, B). Meanwhile, atherosclerotic lesion was assessed in four different vascular sites, including the ascending aorta, descending aorta, thoracic aorta, and abdominal aorta. The results showed that TFA reduced lesions in the above four sites of aorta (Supplementary Figures S1C). Moreover, TFA resulted in significant reduction in the aortic root (Figures 1B,C). Taken together, these data suggests that TFA can retard the atherosclerotic lesion development.

**FIGURE 2:**
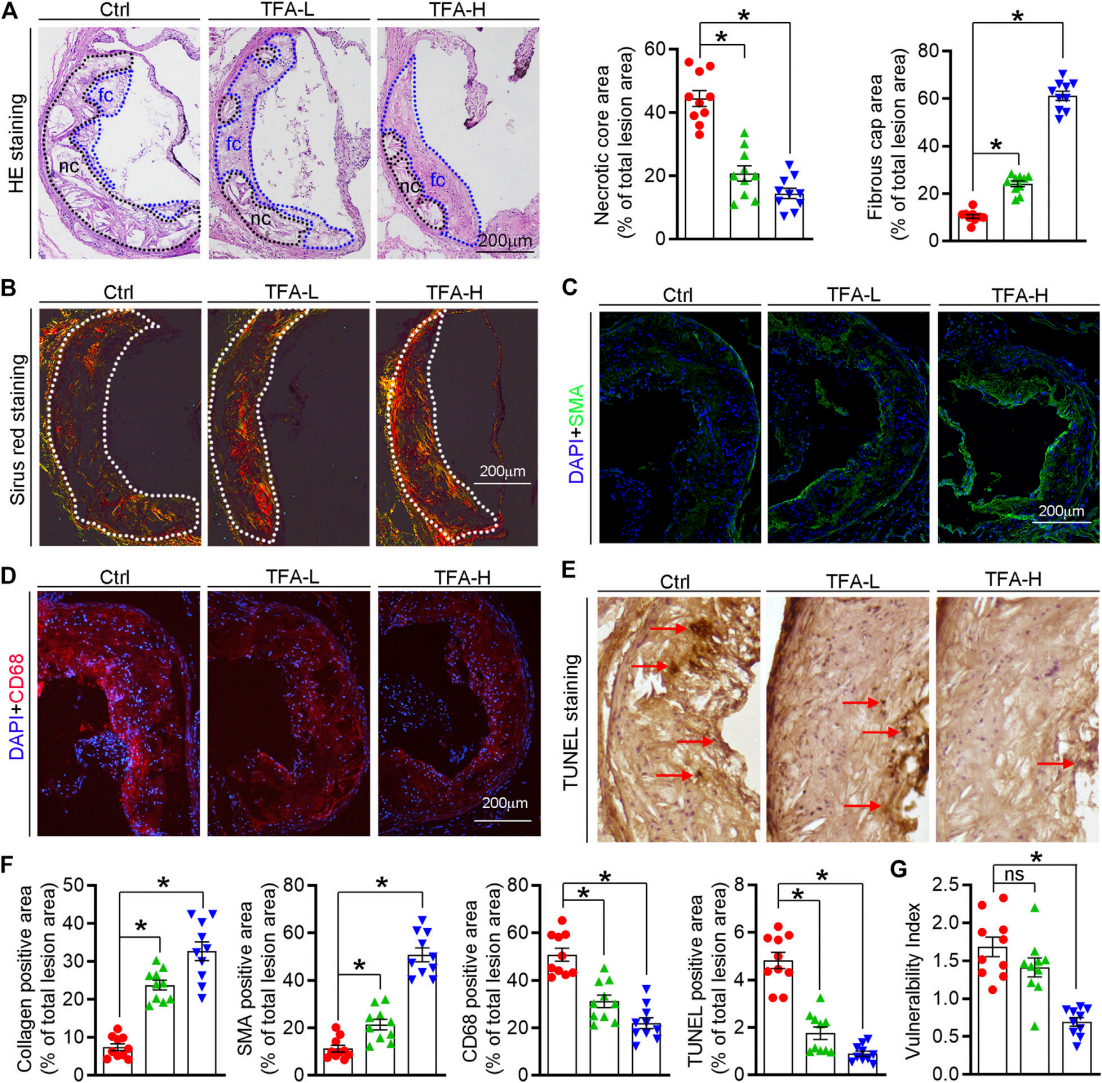
TFA changes the plaque composition and enhances plaque stability. **(A)** Haematoxylin and eosin staining followed by quantitative analysis of sinus lesions, necrotic core area, and fibrous cap area in aortic root cross sections. nc: necrotic cores; fc: fibrous cap, *n* = 10. **(B–F)** Representative photomicrographs of aortic root sections stained with sirus red staining **(B)**, αSMA **(C)**, CD68 **(D)**, and TUNEL staining **(E)** in atherosclerotic plaque followed by the quantification **(F)**, *n* = 10. **(G)** The vulnerability index of plaques was calculated as (macrophage% + lipid staining%)/(SMCs% + collagen fiber%), *n* = 10. Data are presented as mean ± SEM, *p < 0.05, significantly different from control; ns: not significantly different. TFA inhibits foam cell formation by regulating expression of transporters and scavenger receptors that orchestrating cholesterol efflux and uptake.

The high necrotic core size and low fibrous cap area can increase plaque vulnerability and even lead to plaque rupture, which can result in myocardial infarction, stroke, and even sudden death. On aortic cross sections, we found that necrotic core area within the lesions was significantly smaller while the fibrous cap area was larger in TFA treated mice compared to control mice (Figure 2A). In addition, TFA increased the collagen positive area (Figure 2B). Moreover, we observed significant increase in percentages of αSMA^+^ smooth muscle cells and reduction of CD68^+^ foam cells/macrophages in TFA group compared to control group (Figures 2C,D). Dying cells in the plaque can lead to the formation of prothrombotic necrotic core and vulnerable fibrous cap. Therefore, we detected the apoptosis in the plaque by TUNEL staining and immunofluorescent staining with caspase1 antibody; and observed that TFA significantly reduced the cell apoptosis *in situ* (Figure 2E and Supplementary Figures S2). The quantification of Figures 2B–E was represented in Figure 2F. Moreover, the vulnerability index of plaque was reduced by TFA (Figure 2G). Taken together, the data suggests that the stability of atherosclerotic plaque can be enhanced by TFA, by which TFA may reduce the risk of plaque rupture and the following cardiovascular events.

**FIGURE 3:**
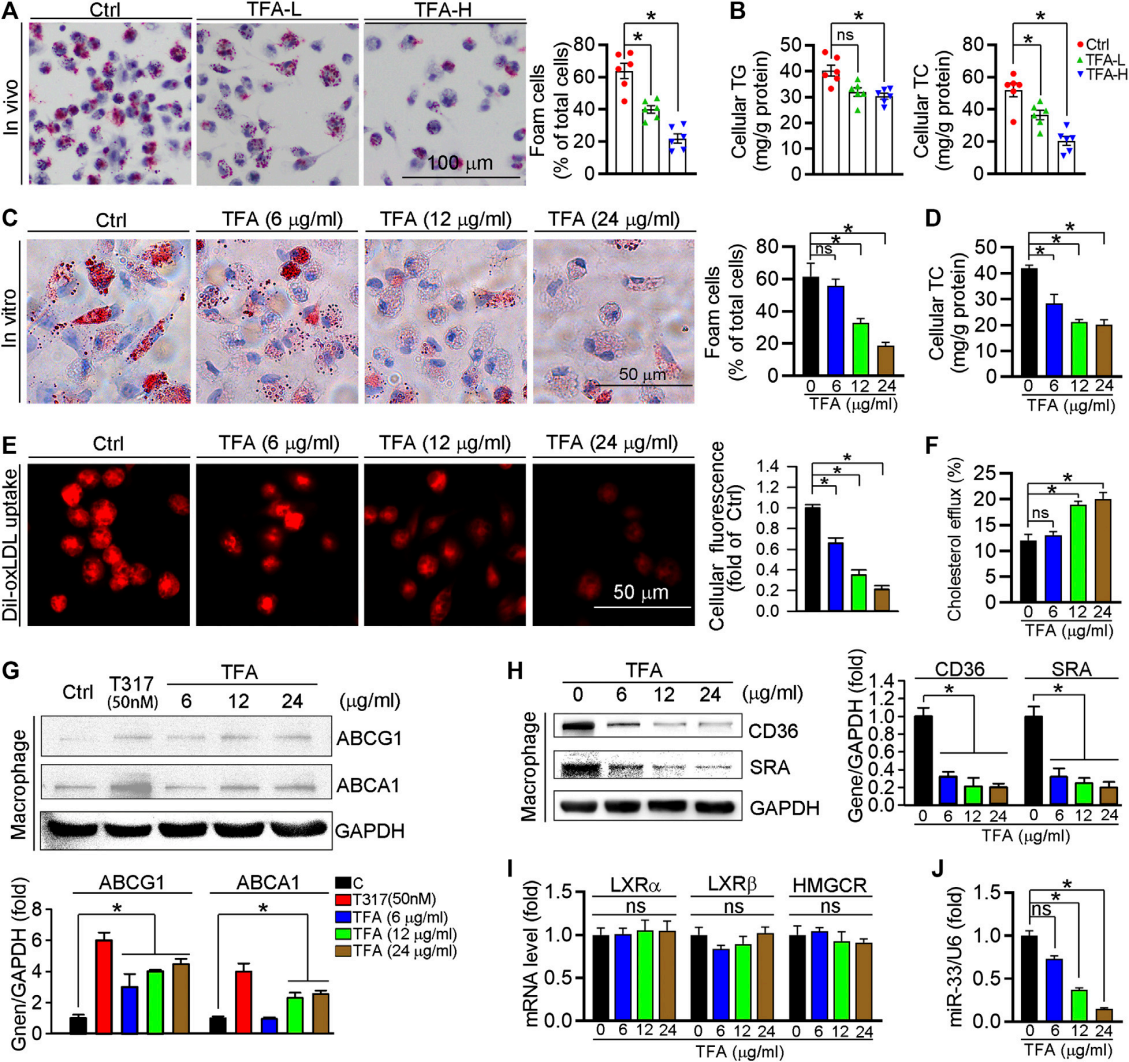
TFA inhibits lipid accumulation and reduces expression of miR-33, CD36 and SRA in macrophage. **(A)** Inhibitory effect of TFA on foam cell formation using Oil Red O staining, *n* = 5. **(B)** Determination of cellular TG and TC in peritoneal macrophage from apoE^−/−^ mice, *n* = 5. **(C)** Inhibitory effect of TFA on lipid accumulation using Oil Red O staining *in vitro* RAW264.7 cells, *n* = 5. **(D)**. Determination of cellular TC in RAW264.7 cells, *n* = 5. **(E)** LDL uptake assay in peritoneal macrophage, *n* = 5. **(F)** Cholesterol efflux assay in peritoneal macrophage, *n* = 5. **(G, H)** After 12 h treatment, expression of ABCA1, ABCG1, CD36, and SRA in peritoneal macrophage was determined by western blot, *n* = 5. **(I)** After 12 h treatment, expression of LXRα, LXRβ, and HMGCR in peritoneal macrophage was determined by q-RT-PCR, *n* = 5. **(J)** After 12 h treatment, expression of miR-33 in peritoneal macrophage was determined by q-RT-PCR, *n* = 5. Data are presented as mean ± SEM, *p < 0.05, significantly different from control; ns: not significantly different. TC: total cholesterol; TG: triglyceride; T317: T0901317, a synthetic ligand of LXR as a positive control. TFA attenuates the inflammatory response in plaque via dual inactivation of NFκB and miR-33.

Foam cell is predominant cells in atherosclerotic plaque, inhibition of which can retard the progression of atherosclerosis. In this study, TFA significantly reduced lipid accumulation in peritoneal macrophages from HFD-treated apoE^−/−^ mice (Figures 3A,B), suggesting the inhibitory effect of TFA on foam cell formation. In addition, TFA markedly reduced the lipid retention in RAW264.7 cells by Oil red O staining (Figure 3C), which was followed by quantification of cellular cholesterol (Figure 3D). Furthermore, we assessed the capacity of cholesterol uptake and efflux, and observed that TFA significantly reduced lipid uptake and enhanced the cholesterol efflux (Figures 3E,F). To delineate the mechanism by which TFA inhibited foam cell formation, we examined the alterations of scavenger receptors (SRA and CD36) and transporters (ABCA1/G1) which are regarded as key mediators in cholesterol homeostasis during foam cell formation. Intriguingly, TFA markedly promoted the ABCA1/G1 expression whereas inhibited the expression of SRA and CD36 (Figures 3G,H). We further disclose the mechanism of TFA on ABCA1/G1 expression. Liver X receptors (LXRs) are the upstream genes of ABCA1/G1. Subsequently, we determined whether TFA could affect the expression of LXRα/β and found that TFA did not affect the expression of LXRα/β in transcriptional level (Figure 3I), indicating that other molecules may mediate the effect of TFA on ABCA1/G1 expression. Moreover, TFA did not change the expression of HMGCR, indicating that TFA did not affect the cholesterol synthesis (Figure 3I). It is well documented that miR-33 is a post-transcriptional regulator of genes involved in cholesterol homeostasis. Noteworthy, miR-33 is a negative regulator of ABCA1/G1. Therefore, we determined whether the TFA-induced ABCA1/G1 expression was involved in miR-33. Intriguingly, TFA significantly reduced the expression of miR-33 (Figure 3J), and miR-33 mimics treatment almost disrupted the promoting effect of TFA on cholesterol efflux (Figure S3), indicating that miR-33 plays a key role in TFA-mediated inhibitory effect on foam cell formation. In addition, we determined the effect of miR-33 mimic on expression of CD36 and SRA1. After transfection of miR-33 mimic in RAW264.7 cells, the expression of CD36 and SRA was evaluated by q-RT-PCR. miR-33 mimic did not affect the expression of either CD36 or SRA (Supplementary Figures S7), indicating that the regulation on CD36 and SRA1 expression by TFA was not miR-33-mediated and other underlying mechanism may exist. Collectively, these results demonstrate that TFA can suppress foam cell formation, mechanistically, through inhibiting scavenger receptor-meditated cholesterol uptake and de-repressing miR-33-mediated restriction on cholesterol efflux.

**FIGURE 4:**
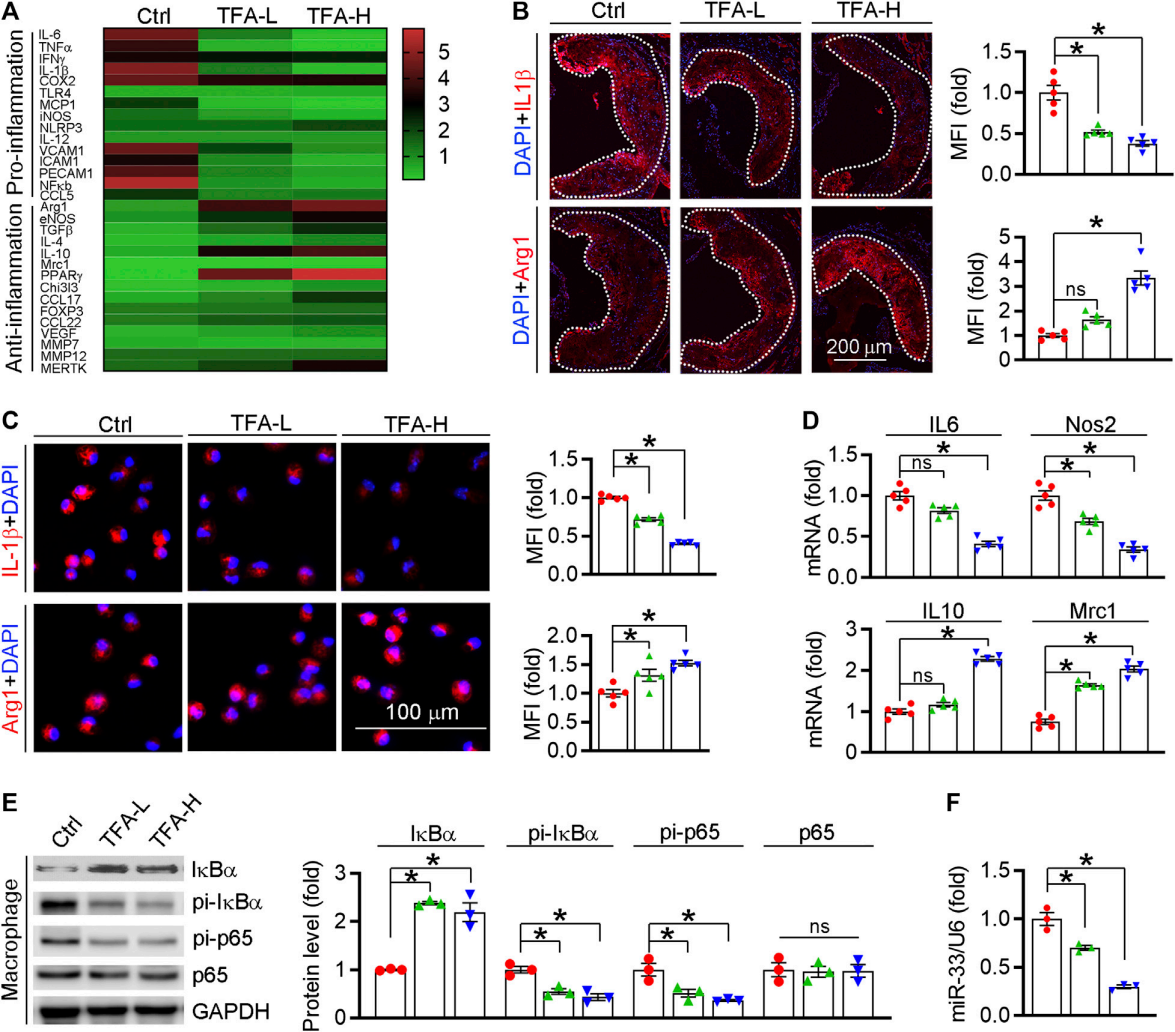
TFA reduces inflammatory response *in vivo*. **(A)** Transcriptional level of atherosclerosis-associated factors was shown in heatmap, *n* = 3. **(B)** Representative pro- and anti-inflammatory factors in the plaque was assessed by immunofluorescent staining, *n* = 5. **(C)** Representative pro- and anti-inflammatory factors in peritoneal macrophage were assessed by immunofluorescent staining, *n* = 5. **(D)** After 12 h treatment, expression of IL10, Nos2, Mrc1, and IL6 in peritoneal macrophage was determined by q-RT-PCR, *n* = 3. **(E)** After 12 h treatment, expression of IκBα, pi- IκBα, pi-p65, and p65 in peritoneal macrophage was determined by western blot, *n* = 3. **(F)** After 12 h treatment, expression of miR-33 in peritoneal macrophage was determined by q-RT-PCR, *n* = 3. Data are presented as mean ± SEM, *p < 0.05, significantly different from control; ns: not significantly different. TFA promotes macrophage phenotypic transition to anti-inflammatory M2 type *in vitro*

Inflammation is an important driver of atherogenesis. Therefore, we assessed the expression of proinflammatory cytokines in aorta; and found that TFA significantly reduced the proinflammatory cytokines whereas promoted the anti-inflammatory cytokines (Figure 4A). We further quantified the levels of representative pro- and anti-inflammatory factors in atherosclerotic lesions and found the expressions of proinflammatory cytokine IL-1β was downregulated while the anti-inflammatory cytokine Arg1 was upregulated in TFA-treated mice (Figure 4B), indicating that TFA ameliorated the inflammation during the lesion formation. Macrophages, as the key mediators of inflammatory response, can affect the progression of atherosclerosis. M1 macrophages are present mainly in unstable plaques and can boost the production of pro-atherogenic inflammatory cytokines, thereby contributing to sustained inflammation and plaque vulnerability. Therefore, we further determined the effect of TFA on macrophage polarization and observed that the peritoneal macrophage from TFA-treated mice are prone to M2 transition but not M1 polarization (Figures 4C,D). To uncover the underlying mechanism of anti-inflammatory effect, we assessed NFκB pathway, the key regulator of inflammation. Noticeable, TFA significantly increased the expression of IκBα and reduced the phosphorylation of IκBα and p65 (Figure 4E), indicating that NFκB pathway was markedly inactivated. Moreover, miR-33 is a post-transcriptional regulator of genes involved in inflammation, and inhibition of which can reduce plaque macrophage inflammation (Distel et al., 2014). Intriguingly, in peritoneal macrophage isolated from TFA-treated mice, expression of miR-33 was markedly reduced (Figure 4F), indicating that the anti-inflammatory effect of TFA may be partially associated with reduction of miR-33 expression. Taken together, we demonstrate that TFA reduced inflammation *in vivo* through inactivation of NFκB and negative regulation of miR-33 expression, by which contributing to its anti-atherogenic function.

**FIGURE 5:**
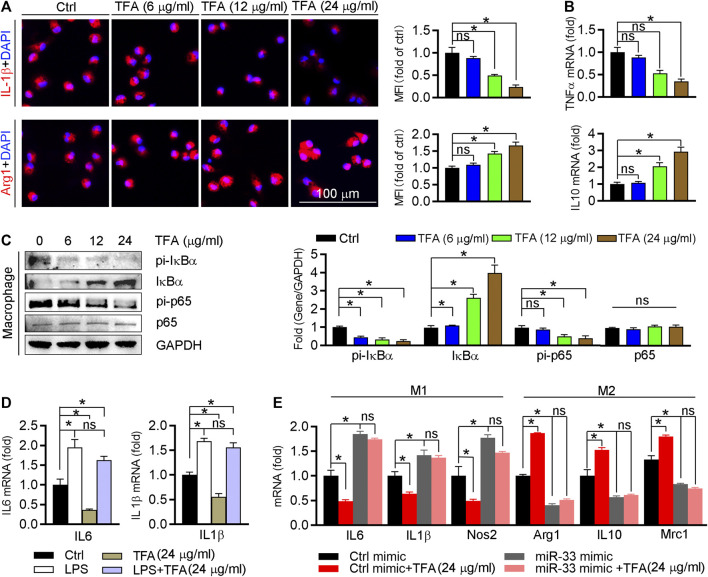
TFA inhibits inflammation and promotes the macrophage M2 transition. **(A)** After TFA treatment in peritoneal macrophage, expression of Arg1 and IL-1β was determined by immunofluorescent staining, *n* = 5. **(B)** After 12 h TFA treatment, expression of TNFα and IL-10 mRNA in peritoneal macrophage was determined by q-RT-PCR, *n* = 5. **(C)** After 12 h TFA treatment, expression of IκBα, pi-IκBα, pi-p65, and p65 in peritoneal macrophage was determined by western blot, *n* = 5. **(D)** After treatment with LPS (100 ng/ml) and TFA, expression of M1 markers in peritoneal macrophage was determined by q-RT-PCR, *n* = 5. **(E)** After 12 h treatment by miR-33 mimics and TFA, expression of M1 or M2 markers in peritoneal macrophage was determined by q-RT-PCR, *n* = 5. Data are presented as mean ± SEM, *p < 0.05, significantly different as indicated; ns: not significantly different. TFA inhibits endothelial activation and monocyte recruitment to endothelial cells.

We further detected effect of TFA on the inflammatory factor generation *in vitro* macrophage. The proinflammatory cytokines IL1β and TNFα were downregulated, whereas the anti-inflammatory cytokines Arg1 and IL10 were upregulated (Figures 5A,B). To uncover the underlying mechanism, we assessed NFκB pathway *in vitro* as we did *in vivo*. Indeed, TFA significantly reduced the activity of NFκB pathway (Figure 5C). To prove the contribution of NFκB to the anti-inflammatory proprieties of TFA, LPS was used to treat peritoneal macrophage to induce the NFκB activation in presence of TFA treatment. After treatment by LPS, the protective effects of TFA on inflammation was significantly abolished (Figure 5D), suggesting that TFA treatment is associated with inactivation of NFκB. Moreover, we further determined whether the anti-inflammatory effect of TFA was associated miR-33. As the data shown, TFA significantly reduced miR-33 expression *in vitro and in vivo* (Figures 3J and 4F), which partially accounted for the anti-inflammatory effect of TFA. Furthermore, we treated macrophages with TFA in presence or absence of miR-33 mimics. Noticeable, TFA promoted the macrophage toward M2 phenotype, which was markedly abolished by miR-33 mimics (Figure 5E), indicating that the regulatory effect on macrophage phenotypic transition is partially miR-33-mediated. Taken together, TFA can inhibit inflammatory *in vitro*, which was associated inactivation of NFκB pathway and negative regulation of miR-33 expression.

**FIGURE 6:**
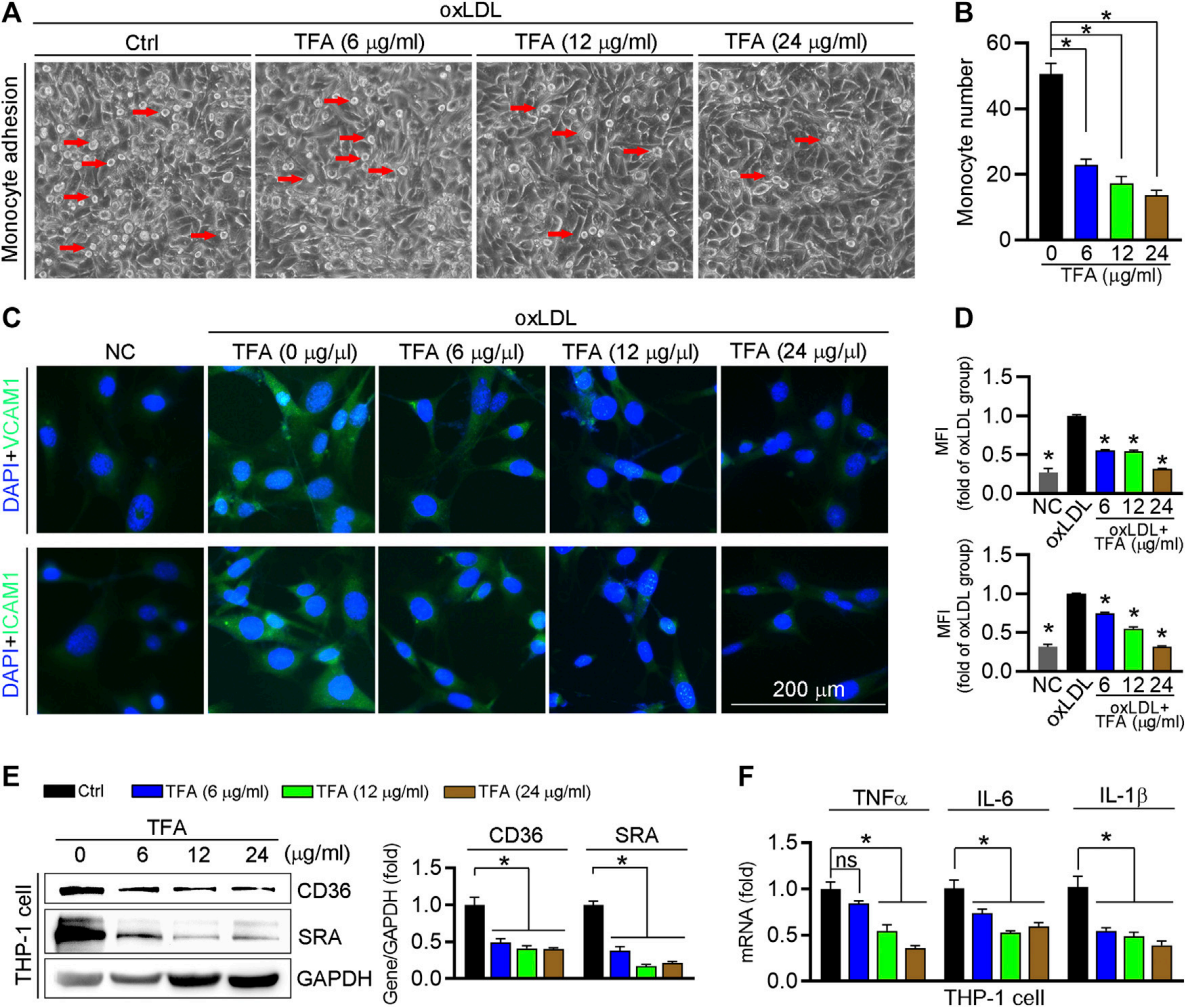
TFA inhibits monocyte adhesion to endothelial cells. **(A, B)** HUVECs in 24-well plates and THP-1 cells were pretreated with oxLDL (100 μg mL^−1^) for 12 h. After incubation with oxLDL, THP-1 cells were added to HUVECs and co-incubated for 1 h. The image of adherent THP-1 cells were captured with a microscope and the number of adherent THP-1 cells was calculated, *n* = 5. **(C, D)** After 12 h TFA treatment in HUVECs, expression of ICAM-1 and VCAM-1 was determined by immunofluorescent staining, *n* = 5. **(E)** Expression of CD36 and SRA protein in THP-1 cells was determined by western blot after 12 h TFA treatment, *n* = 3. **(F)** Expression of proinflammatory cytokines in THP-1 cells was determined by q-RT-PCR after 12 h TFA treatment, *n* = 5. Data are presented as mean ± SEM, *p < 0.05, significantly different as indicated. TFA improves the HFD-induced dyslipidemia in apoE^−/−^ mice and reduces the oxidant stress *in vitro*.

Circulating monocytes are recruited by inflammatory cytokines to the endothelium in the aorta, and then transform into foam cells under LDL or oxLDL stimulation, which contributes to aortic lesions formation. We determined whether TFA could inhibit the monocyte adhesion to endothelial cells and observed that TFA significantly reduced the number of THP-1 cells adhering to human umbilical vein endothelial cells (HUVECs) (Figures 6A,B). Pro-inflammatory cytokines can induce the expression of adhesion molecules in endothelial cells, such as ICAM-1 and VCAM-1, which provide a scaffold for leukocyte migration in endothelial cells. Mechanistically, TFA decreased expression of ICAM-1 and VCAM-1 in HUVEC (Figure 6C). Noteworthy, in THP-1 cells, TFA markedly reduced expression of proinflammatory cytokines, including TNFα, IL1β, and IL-6 (Figure 6F), which contributed to inactivation of the ICAM-1 and VCAM-1 expression in endothelial cells. Due to the important role of miR-33 and NFκB in inflammatory response, we evaluated their role in monocyte adhesion. To prove the contribution of NFκB to inhibition of monocyte recruitment by TFA, LPS was used to treat HUVEC to induce the activation of NFκB in presence of TFA treatment. In addition, HUVEC was treated with miR-33 mimic. After treatment by LPS or miR-33 mimic, the protective effects of TFA on monocyte recruitment was significantly abolished (Supplementary Figures S5), suggesting that TFA treatment is associated with inactivation of NFκB and miR-33. Moreover, adhesion molecules from endothelial cells can bind to specific ligands expressed by monocytes, such as CD36 and SRA, resulting in the increased leukocyte-endothelial interactions (Santiago-García et al., 2003; Trinh-Trang-Tan et al., 2010). Therefore, we further assessed the expression of CD36 and SRA in THP-1 cells and found that both were reduced by TFA (Figure 6E), indicating that the monocyte binding ligand was decreased, which contributed not only to the inhibition of lipid uptake (Figure 3E) but also to the reduction in monocyte adhesion to HUVEC. Collectively, TFA reduced monocyte recruitment to endothelial cells by reducing binding ligands (CD36 and SRA) in monocyte and adhesion molecules (ICAM-1 and VCAM-1) expression in endothelial cells, partially by which exerting the antiatherogenic function.

**FIGURE 7:**
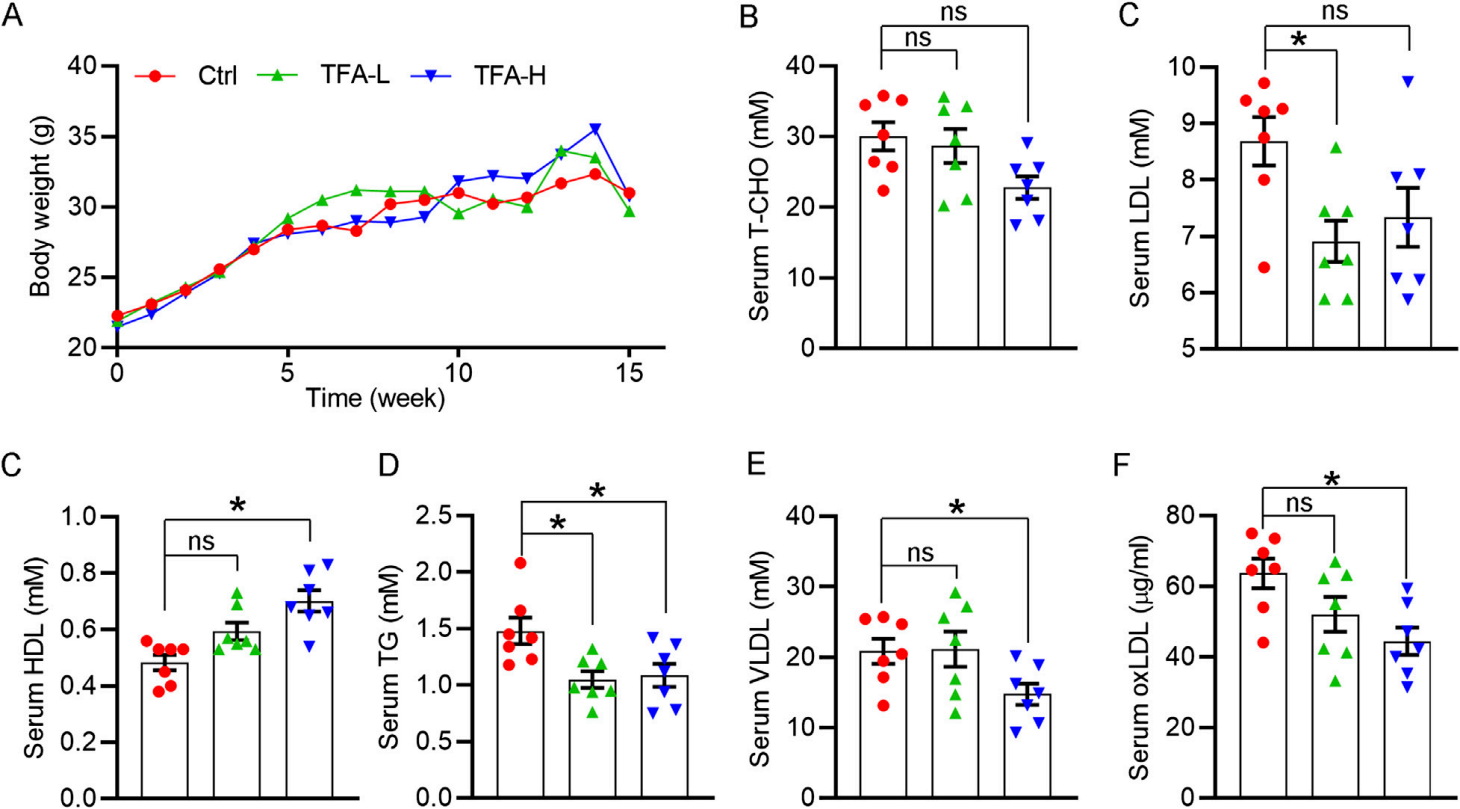
TFA improves the lipid disorder *in vivo* under HFD condition. **(A)** Body weight was recorded weekly. Date are presented as mean ± SEM (*n* = 10). **(B–F)** Serum levels of total cholesterol (T-CHO), LDL- and HDL-C, VLDL, TG (mM) and oxLDL (μg/ml) were determined by biochemical analyzer or ELISA assay. Date are presented as mean ± SEM (*n* = 7), *p < 0.05, significantly different from control; ns: not significantly different. TFA ameliorates the HFD-induced hepatic steatosis in apoE^−/−^ mice.

Lipid dysfunction is a critical contributor to atherosclerosis development. The influx of LDL into the arterial intima, the site of atherogenesis, is closely associated with their plasma concentration because high concentrations of LDL lead to higher LDL uptake by macrophages. In addition, infiltrated LDL are oxidized to turn into highly atherogenic form, such as ox-LDL. Macrophages ingest the modified LDL particles via scavenger receptors and thereby transformed into foam cells. In contrast, HDL is considered antiatherogenic lipoproteins because it can initiate the reverse cholesterol transport, by which transfering cholesterol from foam cells to the liver, and ultimately to the gut for excretion. In this study, the body weight of mice was not changed by TFA (Figure 7A). Of note, TFA significantly reduced level of total cholesterol (T-CHO), LDL, TG, and VLDL, whereas increased the HDL level (Figures 7B–E). In addition, we quantified the level of ox-LDL, a modified LDL that contributed to atherogenesis, and observed that ox-LDL level was downregulated in TFA-treated mice compared with control mice (Figure 7F), suggesting that the atherogenic form of LDL was reduced and oxidant stress may be attenuated by TFA. Therefore, we further determined the cellular ROS levels by DCF staining. Intriguingly, DCF fluorescent intensity was reduced by TFA in dose-dependent manner (Supplementary Figures S6), indicating that TFA reduced the production of cellular ROS. Collectively, TFA improved the lipid profile and restrained the transformation of LDL to atherogenic form during the atherosclerosis development, partially by which TFA exerted the anti-atherogenic function.

**FIGURE 8:**
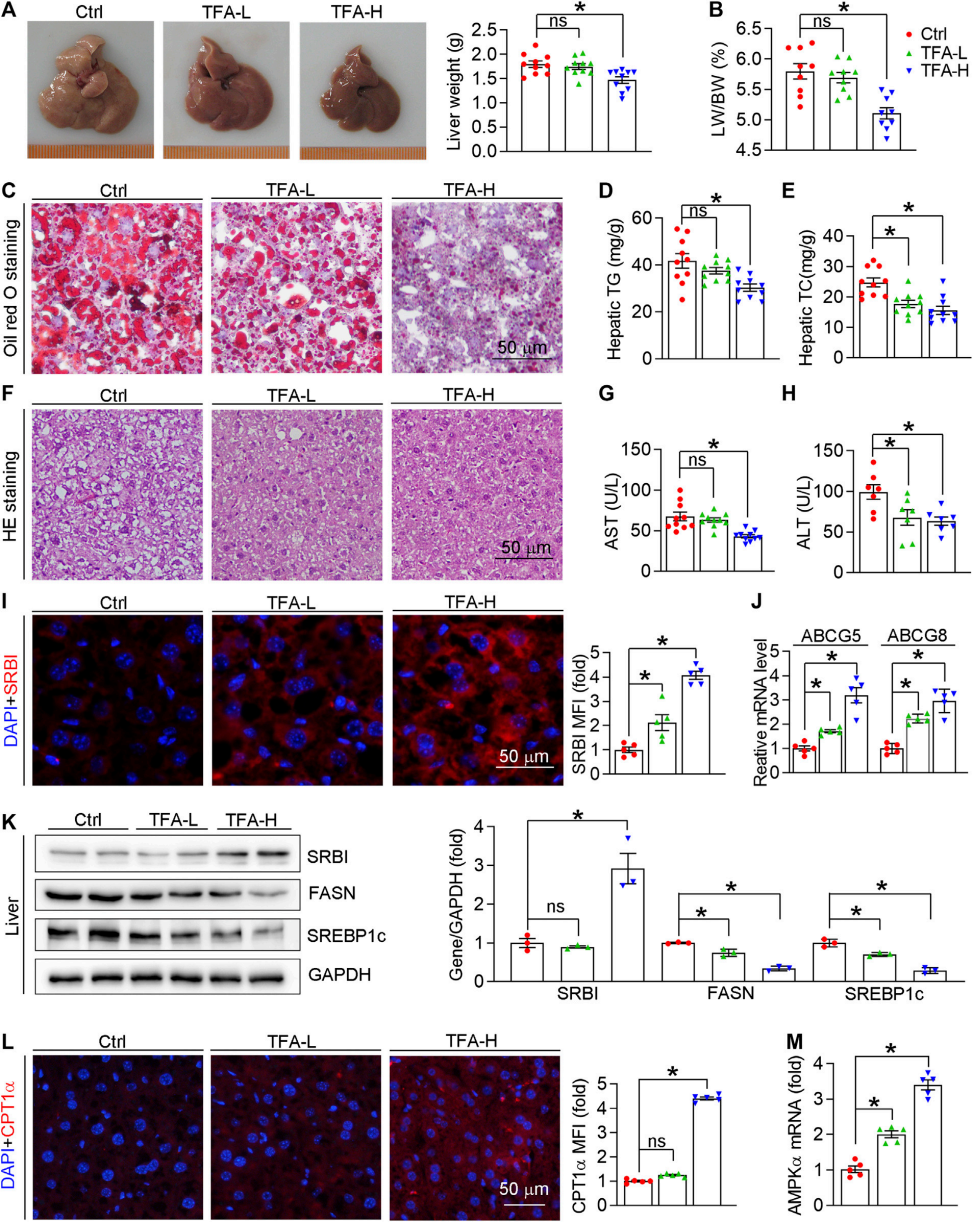
TFA ameliorates hepatic steatosis *in vivo*. **(A)** Liver photos and weight, *n* = 10. **(B)** Ratio of liver to body weight, *n* = 10. **(C)** Oil Red O staining on liver frozen sections, *n* = 10. **(D, E)** Quantitative analysis of hepatic TG and TC. **(F)** Haematoxylin and eosin staining of liver frozen sections, *n* = 10. **(G,H)** Serum levels of AST (*n* = 10) and ALT (*n* = 7). **(I)** Expression of SRBI in liver was determined by immunofluorescent staining, *n* = 5. **(J)** Expression of ABCG5 and ABCG8 in liver was determined by q-RT-PCR, *n* = 5. **(K)** Expression of SRBI, FASN, and SREBP1c in liver was determined by western blot, *n* = 3. (**L**) Expression of CPT1α in liver was determined by immunofluorescent staining, *n* = 5. (**M**) Expression of AMPKα in liver was determined by q-RT-PCR, *n* = 5. Data are presented as mean ± SEM, *p < 0.05, significantly different as indicated.

Hepatic steatosis facilitates atherogenesis because the liver plays an important role in lipid metabolism, such as RCT, lipid synthesis, and fatty acid oxidation (Patel and Siddiqui, 2019; Stols-Gonçalves et al., 2019; Jiang et al., 2020). As shown in Figure 8A, liver color and weight were changed to almost normal condition, which was accompanied by reduced ratio of liver weight to body weight (Figure 8B). Moreover, H&E and Oil Red O staining revealed that hepatic steatosis was attenuated in TFA-treated mice compared to control mice (Figures 8C,F). Furthermore, TFA significantly reduced the hepatic TC and TG content (Figures 8D,E), indicating that hepatic steatosis was attenuated by TFA. Hepatic steatosis can lead to the liver injury. Noticeable, chronic treatment with TFA did not cause hepatic toxicity as measured by plasma AST and ALT enzyme levels. In contrast, TFA markedly reduced the HFD-induced liver injury, which was shown by the reduced levels of AST and ALT (Figures 8G,H). To further determine the mechanism of TFA on hepatic lipid metabolism, we assessed the expression of genes involved lipid oxidation and genesis. Intriguingly, TFA markedly promoted the expression of SRBI, the gene in charge of cholesterol uptake in liver; and simultaneously promoted the expression of ABCG5/G8, the genes responsible for cholesterol transportation to intestine (Figures 8I,J), by which enhanced the RCT and then reduced the hepatic retention of cholesterol. In addition, TFA significantly reduced expression of genes responsible for lipid genesis, including FASN and SREBP1c (Figure 8K), indicating that TFA inhibited hepatic lipid synthesis. Consistent with this observation, the protein or mRNA expression of genes involved in fatty acid oxidation (AMPKα and CPT1α) were upregulated in TFA-treated mice (Figures 8L,M), which indicated that TFA promoted the lipid consumption. Taken together, TFA attenuated the hepatic steatosis by promoting RCT, enhancing fatty acid oxidation, and downregulating lipid synthesis, by which improving the lipid metabolism and thereby partially accounting for its antiatherogenic function.

## Discussion

Atherosclerosis, a major pathogen of coronary heart disease (CHD), is closely associated with lipid disorder and chronic inflammation. TFA is the flavone component from *A. membranaceus* which is the long-term used traditional Chinese medicine for CHD treatment in clinic. Noticeable, the current study showed that higher intake of flavone was associated with a lower risk of CHD (Ma L. et al., 2020). In this study, we determined whether TFA could inhibit atherosclerosis, and attempted to uncover the underlying mechanism. Intriguingly, TFA treatment substantially reduced the atherosclerotic development, enhanced the plaque stability, and reduced monocyte recruitment, which may be attributed to the inhibition of lipid disorder and inflammation. Mechanistically, we found that miR-33 is a critical signaling mediator of TFA on promoting ABCA1/G1 expression as well as inhibiting inflammation in macrophages, which coordinated the anti-inflammatory effect of TFA by inactivating NFκB signaling pathway.

Increased vulnerability is prone to plaque rupture, which can result in severe cardiovascular event (Seneviratne et al., 2017). Of note, composition of plaque markedly affects the lesion stability (Naghavi et al., 2003). In this study, TFA significantly reduced atherosclerotic plaque size and promoted a more favorable plaque composition with increased fibrous cap, plaque collagen and SMC content; and reduced necrotic core area and macrophage accumulation, indicating that TFA significantly reduced the plaque vulnerability. Moreover, TFA markedly promoted the expression of ABCA1/G1 and inhibited the CD36 and SRA expression (Figures 3G,H), by which enhancing cholesterol efflux, reducing cholesterol uptake and thereby inhibiting foam cell formation. However, as the upstream regulatory gene of ABCA1/G1, liver X receptor (LXR) was not activated by TFA (Figure 3I). Moreover, the key gene for cholesterol synthesis, HMGCR, was also not affected by TFA (Figure 3I). These results indicated that other molecule may mediate the TFA-induced of ABCA1/G1 expression.

To gain further insight into the effect of TFA on ABCA1/G1 expression, we investigated the mechanism. Noticeable, miRNAs, a group of small endogenous non-coding RNAs, can regulate gene expression at posttranscriptional levels. Functionally, miRNAs can serve as important regulators of atherogenic process, such as cellular adhesion, lipid uptake and efflux, and generation of inflammatory mediators (Feinberg and Moore, 2016). Therefore, we hypothesized that miRNAs may participate in the protective effect of TFA on lipid metabolism and inflammation. Noticeable, miR-33 is a negative regulator of ABCA1/G1 and thereby promoting foam cell formation via inhibiting macrophage cholesterol efflux (Rayner et al., 2011b). We further postulated that the enhancement of ABCA1/G1 and the inhibition of inflammation by TFA may be through suppression of miR-33, by which removing the restriction on expression of ABCA1/G1. To test the hypothesis, we evaluated miR-33 expression *in vitro* and *in vivo*, and found that miR-33 was reduced by TFA (Figure 3J), which accounted for the upregulation of ABCA1/G1 expression.

Atherosclerosis is characterized with the predominance of an M1 macrophage phenotype within the plaque, whereas plaques undergoing regression are rich in M2 macrophages (Lutgens et al., 2019; Tunon et al., 2019). Intriguingly, in this study, TFA significantly inhibited M1 macrophage polarization, whereas promoted M2 macrophage phenotype *in vivo* and *in vitro*, as indicated by the reduced levels of proinflammatory cytokines and increased anti-inflammatory cytokines (Figures 4A–D and 5A). NFκB signaling pathway plays important role in regulating macrophage polarization and inflammation (Song et al., 2019). In addition, miR-33 can sustain the inflammatory M1 macrophage phenotype, and inhibition of which can reduce plaque inflammation (Distel et al., 2014; Ouimet et al., 2015). Therefore, we determined whether the anti-inflammatory effect of TFA was involved in NFκB and miR-33. We assessed not only the activity of NFκB pathway but also the expression of miR-33, and observed that TFA markedly inactivated the NFκB activity (Figures 4E and 5C) and reduced the expression of miR-33 (Figure 4F). Intriguingly, miR-33 mimic or LPS almost abolished the effect of TFA on macrophage inflammation (Figure 5D, E). These results indicated that anti-inflammatory effect of TFA may be attributed to dual suppression of miR-33 and NFκB pathway.

Atherogenic endothelial activation enhances circulating monocytes adhesion, and especially in the context of hypercholesterolemia. These monocytes uptake excessive lipid to drive early plaque formation (Tabas et al., 2015). In the monocyte attachment assay, TFA markedly suppressed the monocyte recruitment to HUVECs, and mechanistically reduced the expression of VCAM-1 and ICAM-1 (Figures 6A–D), the major molecules that expressed in the activated ECs (Gerhardt and Ley, 2015), indicating that TFA can attenuate the endothelial activation and the following monocyte adhesion. Moreover, TFA significantly reduced the HFD-induced the lipid disorder (Figures 7A–F), which favored the reduction in cellular lipid accumulation in monocyte and thereby retarding the initiation and development of atherosclerosis.

Liver is a critical organ that regulates lipid metabolism, which mainly determines the cholesterol metabolism and lipid profile in serum, thereby affecting the atherogenesis. Despite no significant differences were observed in body weight over the 16 weeks of HFD along with TFA feeding, the serum lipid profile was significantly improved by TFA (Figure 7). Mechanistically, SREBP-1c can activate transcription of genes involved in lipid synthesis, such as fatty acid synthase (FASN) (Shimano, 2009). Noteworthy, in the liver, TFA markedly damped the expression of SREBP-1c and FASN while increased the expression of CPT1α and AMPKα (Figures 8K–M), indicating that lipid synthesis was decreased, and fatty acid oxidation (FAO) was increased. Noticeable, miR-33, an intronic miRNA that co-expressed with its host gene SREBP1 (Najafi-Shoushtari et al., 2010), can balance cellular lipid levels by increasing genes that oppose SREBP-regulated pathways, including those involved in cholesterol efflux and FAO. For instance, in non-human primate model, miR-33 antagonism increased FAO and reduced fatty acid synthesis (Rayner et al., 2011a). In this study, hepatic miR-33 expression was reduced by TFA (Supplementary Figures S4), which may be attributed to repression of SREBP1 (Figure 8K). In addition, scavenger receptor class B member 1 (SR-BI) is key molecule for RCT, loss of which in people are associated with increased cardiovascular risk (Zanoni et al., 2016). After ABCA1/G1-mediated cholesterol efflux to HDL, mature HDL can directly deliver cholesterol to the liver via SR-BI, by which mediates RCT and are thereby anti-atherogenic (Rader et al., 2009; Rosenson et al., 2012; Shen et al., 2018). In this study, TFA significantly enhanced SRBI expression (Figures 8I,K), which may promote RCT and thereby inhibit atherogenesis. Additionally, the TFA-induced promotion in efflux capacity was significantly correlated with plasma HDL concentrations (Figure 7C). Moreover, the biliary cholesterol excretion rate is mainly dependent on the expression of ABCG5/G8 (Nijstad et al., 2011; Lee-Rueckert et al., 2016); and it should be noted that the RCT rate in the mice can be promoted by increasing the hepatic expression of ABCG5/G8 (Escolà-Gil et al., 2011). In the present study, TFA markedly increased the expression of the ABCG5/G8 (Figure. 8J), indicating that TFA may contribute to the cholesterol excretion into bile and thereby increasing RCT, by which inhibiting the foam cell formation in the atherogenic plaque.

This study explored a broad variety of potential downstream mechanisms that could be associated with TFA effects. However, the limitation of the study is that upstream mechanisms need to be further investigated. Oxidant stress is a factor of atherogenesis (Förstermann et al., 2017). Previous study has reported the antioxidant effect of TFA *in vitro* (Xu et al., 2014). In our study, TFA reduced the oxLDL level in the serum (Figure 7F) and reduced the ROS generation *in vitro* (Supplementary Figures S6), indicating that oxidant stress may be attenuated by TFA. These data indicate that TFA may react with reactive oxygen species associated with atherosclerosis, which then triggers the observed downstream mechanisms.

## Conclusion

Taken together, TFA can attenuate the atherosclerotic development via inhibiting foam cell formation and inflammation, which is through negative regulation of miR-33, CD36, and SRA expression; and NF-κB pathway. Simultaneously, TFA markedly ameliorates hepatic steatosis via improving the hepatic lipid metabolism, and thereby reduces proatherogenic lipid disorder, which is through promoting reverse cholesterol transport, enhancing fatty acid oxidation, and downregulating lipid synthesis. Therefore, TFA may act as a very promising anti-atherosclerotic drug by activating multiple signaling pathways that regulating lipid metabolism and inflammation.

## Data Availability Statement

The original contributions presented in the study are included in the article/Supplementary Material, further inquiries can be directed to the corresponding authors.

## Ethics Statement

The animal study was reviewed and approved by Ethics Committee of Tianjin University of Traditional Chinese Medicine.

## Author Contributions

JZ, SY, and YH conducted the experiment; CM wrote the article; JS, YS, ZW, KF, JZ, XY, and HZ offered the advice; JM and GF designed the experiment.

## Funding

This work was supported by Grants from National Major Scientific and Technological Special Project for “Significant New Drugs Development” (2019ZX09201005-007-001); National Natural Science Foundation of China (81774050, 82003747, 82000824); Tianjin Outstanding Youth Science Foundation (17JCJQJC46200); Natural Science Foundation of Tianjin (19JCQNJC12600, 20JCQNJC00260); Research project of Tianjin education commission (2019KJ044); Training Program Foundation for Innovative Research Team of Higher Education in Tianjin during the 13th Five-Year Plan Period (No. TD13-5050).

## Conflict of Interest

The authors declare that the research was conducted in the absence of any commercial or financial relationships that could be construed as a potential conflict of interest.

## Abbreviations

Arg1, arginase 1; CPT1α, carnitine palmitoyl transferase 1 alpha; CXCL1, C-X-C motif chemokine ligand 1; IFN-γ, Interferon gamma; Mgl2, macrophage lectin 2; Mrc1, mannose receptor C-type 1; NOS2, nitric oxide synthase 2; SREBP1c, sterol regulatory element-binding protein 1c; TGF-β1, transforming growth factor β1; FASN, fatty acid synthase; SR-BI, scavenger receptor class B type 1; RCT, reverse cholesterol transport; IL, interleukin; TNFα, tumor necrosis factor α; iNOS, inducible nitric oxide synthase; GAPDH, glyceraldehyde-3-phosphate dehydrogenase; COX2, prostaglandin-endoperoxide synthase 2; TLR4, toll like receptor 4; MCP-1, monocyte chemotactic protein 1; NLRP3, NLR family pyrin domain containing 3; VCAM-1, vascular cell adhesion molecule 1; ICAM-1, intercellular adhesion molecule 1; PECAM1, platelet and endothelial cell adhesion molecule 1; NF-κB, nuclear factor kappa B; CCL, C-C motif chemokine ligand; eNOS, endothelia NO synthase; PPARγ, peroxisome proliferator activated receptor gamma; Chi3I3, chitinase-like 3; FOXP3, fork head box P3; VEGF, vascular endothelial growth factor; MMP, matrix metallopeptidase; MERTK, MER proto-oncogene tyrosine kinase; HMGCR, 3-hydroxy-3-methylglutaryl-CoA reductase; LXRα/β, Liver X receptor α/β
