# Supplementary material for: Astragalus Flavone Ameliorates Atherosclerosis and Hepatic Steatosis Via Inhibiting Lipid-Disorder and Inflammation in apoE−/− Mice
Source: Front Pharmacol. 2020 Dec 14;11:610550. doi: 10.3389/fphar.2020.610550 (PMC7768082; doi:10.3389/fphar.2020.610550)
Supplement: Supplementary file 1 [file datasheet1.docx]

**Supplementary materials**

**Astragalus flavone ameliorates atherosclerosis and hepatic steatosis via inhibiting lipid-disorder and inflammation in apoE^-/-^ mice**

Chuanrui Ma^1,2#^, Jing Zhang^1,2#^, Shu Yang ^4,5#^, Yunqing Hua^1,2^, Jing Su^3^, Yuna Shang^6^, Zhongyan Wang^7^, Ke Feng^6^, Jian Zhang^8^, XiaoxiaoYang^9^, Hao Zhang^1,2^, Jingyuan Mao^1,2*^, Guanwei Fan ^1,2 *^

^1^First Teaching Hospital of Tianjin University of Traditional Chinese Medicine, Tianjin, China; ^2^Tianjin Key Laboratory of Translational Research of TCM Prescription and Syndrome, Tianjin, China; ^3^Tianjin State Key Laboratory of Component-based Chinese Medicine，Tianjin University of Traditional Chinese Medicine, Tianjin, China. ^4^Department of Endocrinology, The Second Clinical Medical College, Jinan University (Shenzhen People's Hospital), Shenzhen, China; ^5^Integrated Chinese and Western Medicine Postdoctoral Research Station, Jinan University, Guangzhou, China; ^6^College of Life Sciences, Nankai University, Tianjin, China; ^7^Tianjin Key Laboratory of Radiation Medicine and Molecular Nuclear Medicine, Institute of Radiation Medicine, Chinese Academy of Medical Sciences & Peking Union Medical College, Tianjin, China; ^8^Department of Pharmacology, College of Basic Medical Sciences, Tianjin Medical University. Tianjin, China; ^9^Key Laboratory of Metabolism and Regulation for Major Diseases of Anhui Higher Education Institutes, College of Food and Biological Engineering, Hefei University of Technology, Hefei, China

^#^These authors contributed equally to this article.

*Correspondence should be addressed to:

Guanwei Fan, PhD; Jingyuan Mao, PhD

Tianjin Key Laboratory of Translational Research of TCM Prescription and Syndrome, Tianjin University of Traditional Chinese Medicine, Tianjin, China

No.88, Chang Ling Road, Li Qi Zhuang Jie, Xi Qing District, Tianjin, P.R. China; Tel:86-22-27987795; Fax:86-22-27987795; E-mail: guanwei.fan@tjutcm.edu.cn


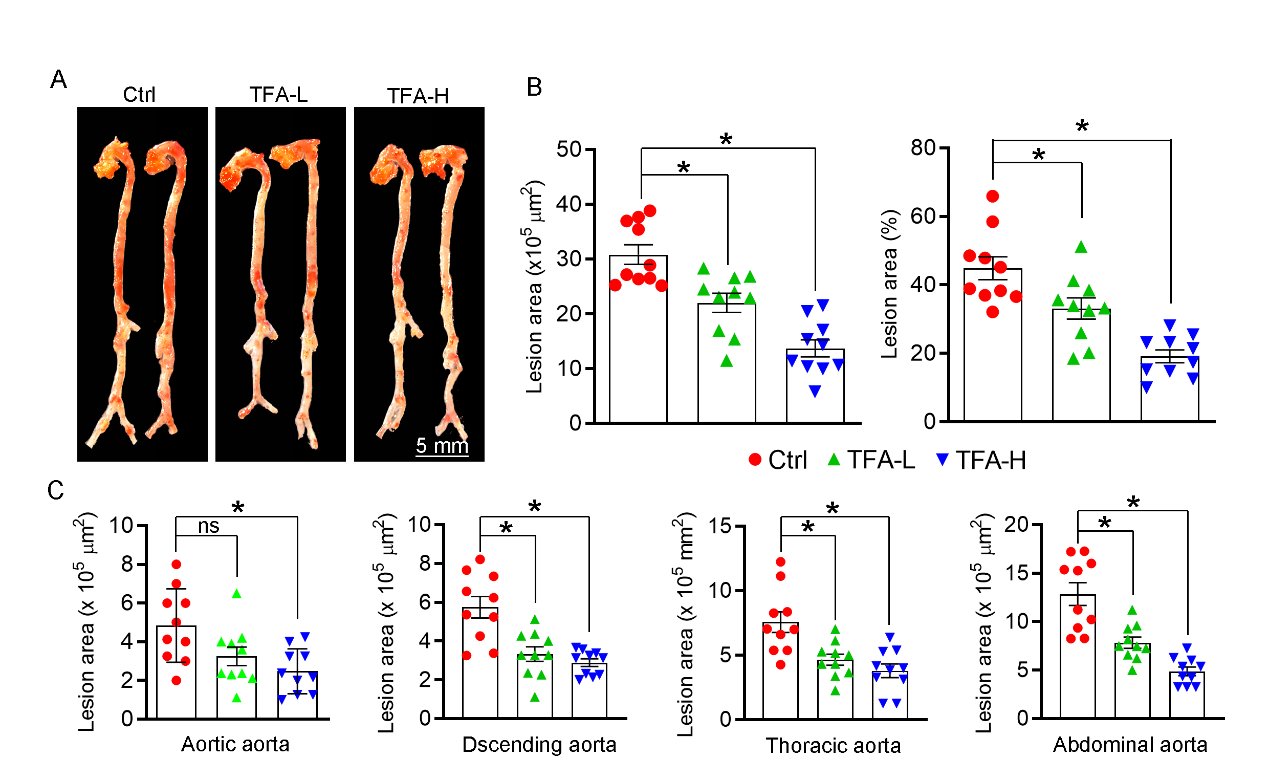


**Figure S1. TFA reduces *en face* aortic lesions in apoE^-/-^ mice.** (**A-C**) Representative *en face* morphometric images of total aortic lesion area and calculated whole aortic atherosclerosis. Lesion areas were expressed as μm^2^ or the ratio of lesion area to total area of aorta, n=10. Data are presented as mean ± SEM, ns, not significantly different.


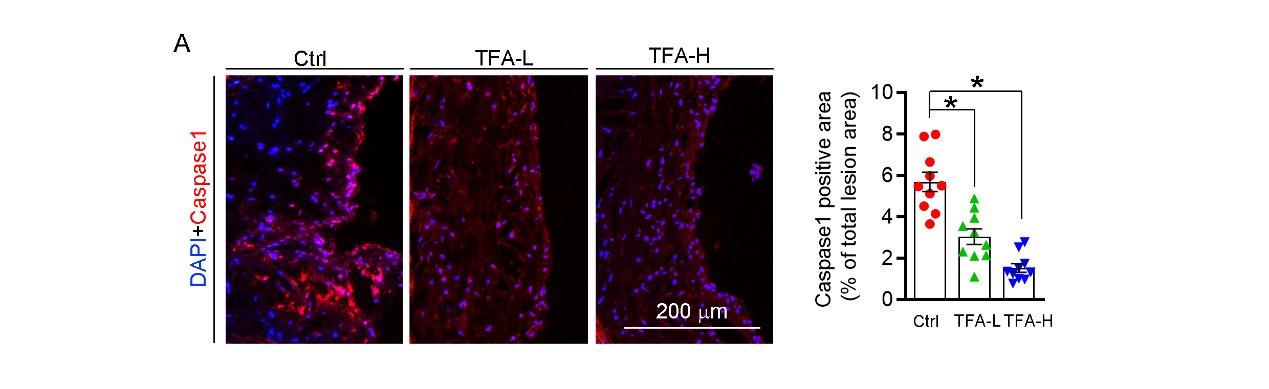


**Figure S2. TFA reduces caspase 1 staining in aortic lesions in apoE^-/-^ mice.** (**A**) Representative photomicrographs of aortic root sections of immunofluorescent staining with caspase1 antibody in atherosclerotic plaque followed by the quantification, n=10.


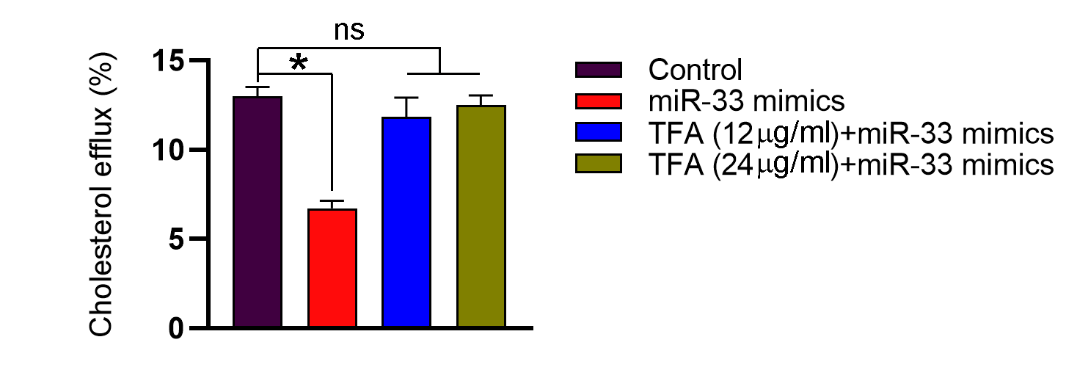


**Figure S3. miR-33 mimics abolished the effect of TFA on cholesterol efflux.** Macrophages were seeded into plate and cultured to 60% confluence and then transfected with miR-33 mimics in presence or absence of TFA. After treatment, the level of cholesterol efflux was evaluated as we did in the manuscript, n=5. Data are presented as mean ± SEM, ns, not significantly different.

**
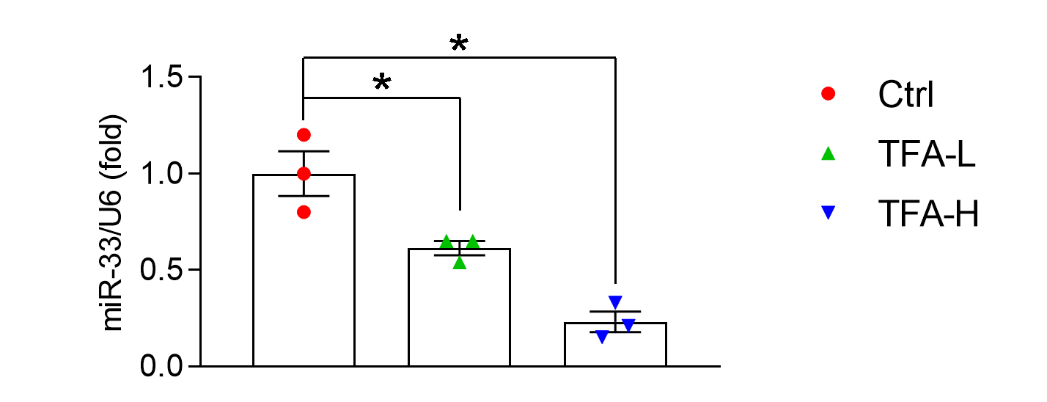
**

**Figure S4. Hepatic miR-33 expression was assessed.** After treatment as indicated in Figure 1, the expression level of miR-33 in liver was evaluated by q-RT-PCR, n=5. Data are presented as mean ± SEM, ns, not significantly different.


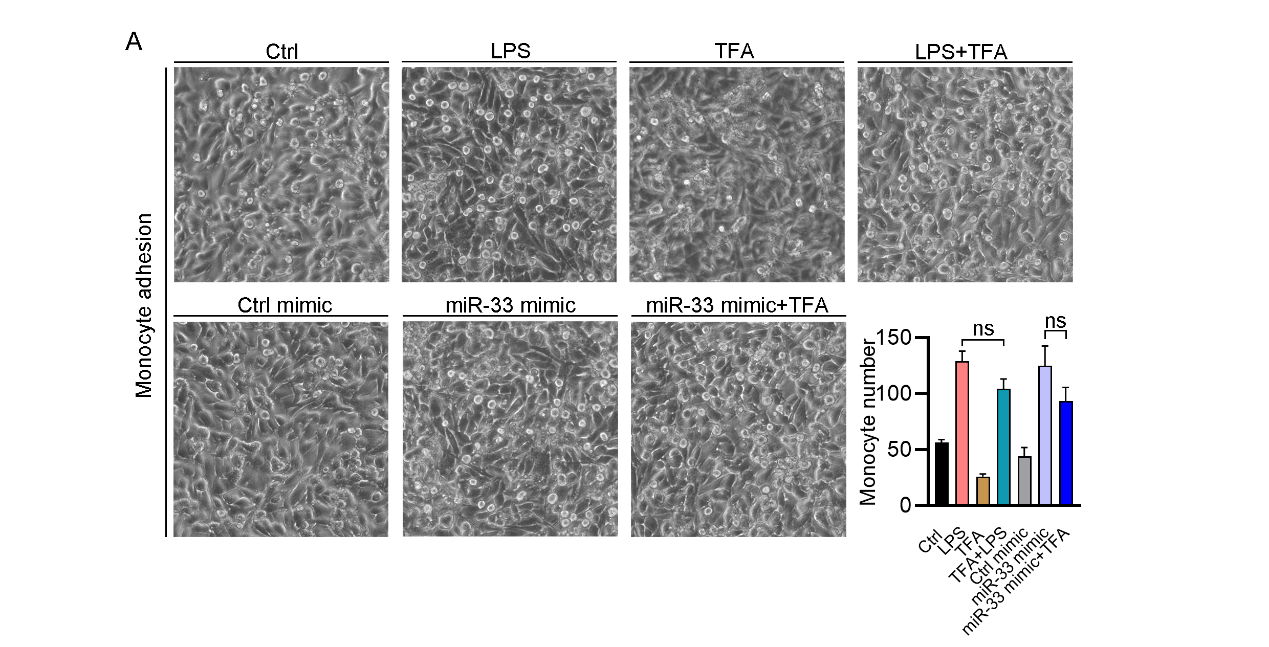


**Figure S5. LPS or miR-33 mimic abolished the inhibitory effect of TFA on monocyte adhesion.** (A) HUVECs in 24-well plates and THP-1 cells were pretreated with LPS (100 ng mL^-1^) for 2 h or transfected with miR-33 mimic (50 nM) in presence of TFA treatment. After treatment, THP-1 cells were added to HUVECs and co-incubated for 1 h. The image of adherent THP-1 cells were captured with a microscope and the number of adherent THP-1 cells was calculated, n=5. Data are presented as mean ± SEM, ns, not significantly different.


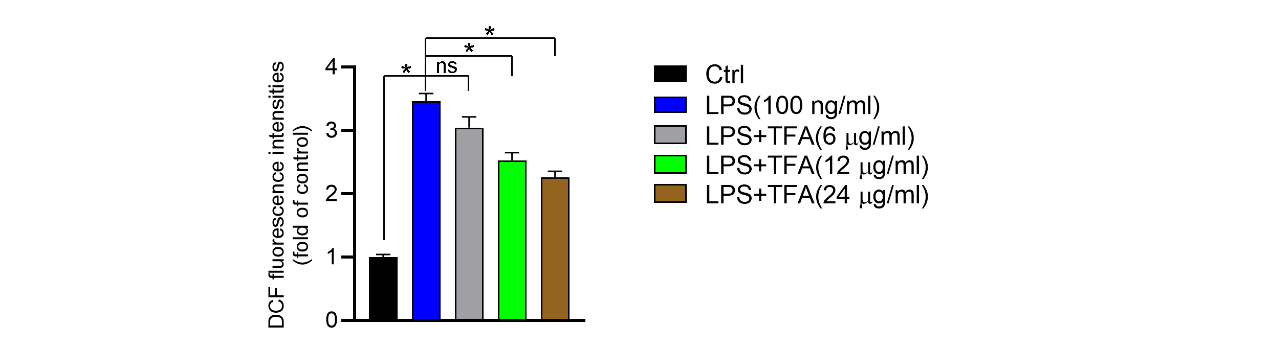


**Figure S6. TFA reduces cellular ROS levels.** Cellular ROS levels in RAW264.7 were determined by DCF staining, n=5. Data are presented as mean ± SEM, *P<0.05, significantly different as indicated; ns, not significantly different.


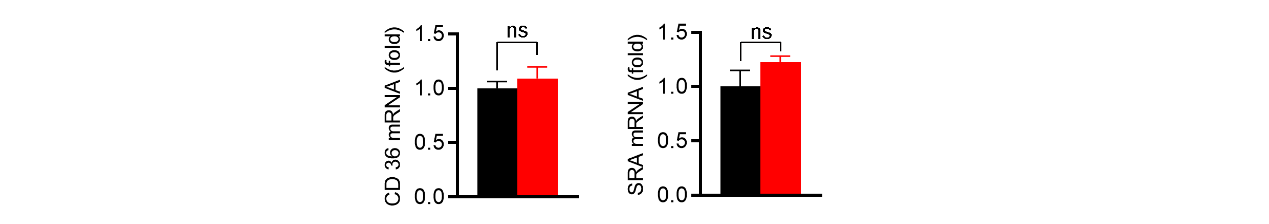


**Figure S7. miR-33 mimic did not affect the expression of CD36 and SRA.** After transfection of miR-33 mimic in RAW264.7 cells, the expression of CD36 and SRA was evaluated by q-RT-PCR, n=3. Data are presented as mean ± SEM, ns, not significantly different.

**Table S1. Sequences of primers for q-RT-PCR**

| **GENE** | **Forward** | **Backward** |
| --- | --- | --- |
| ***Mus IL-1β*** | GCAACTGTTCCTGAACTCAACT | ATCTTTTGGGGTCCGTCAACT |
| ***Mus IL-6*** | CCAAGAGGTGAGTGCTTCCC | CTGTTGTTCAGACTCTCTCCCT |
| ***Mus TNFα*** | GACGTGGAACTGGCAGAAGAG | TTGGTGGTTTGTGAGTGTGAG |
| ***Mus iNOS*** | GTTCTCAGCCCAACAATACAAGA | GTGGACGGGTCGATGTCAC |
| ***Mus GAPDH*** | AGGTCGGTGTGAACGGATTTG | TGTAGACCATGTAGTTGAGGTCA |
| ***Mus IFNγ*** | ATGAACGCTACACACTGCATC | CCATCCTTTTGCCAGTTCCTC |
| ***Mus COX2*** | CTGACCCCCAAGGCTCAAAT | TCCATCCTTGAAAAGGCGCA |
| ***Mus*** ***TLR4*** | ATGGCATGGCTTACACCACC | GAGGCCAATTTTGTCTCCACA |
| ***Mus MCP-1*** | TTAAAAACCTGGATCGGAACCAA | GCATTAGCTTCAGATTTACGGGT |
| ***Mus*** ***NLRP3*** | TGTGAGAAGCAGGTTCTACTCT | GACTGTTGAGGTCCACACTCT |
| ***Mus IL-12*** | TGGTTTGCCATCGTTTTGCTG | ACAGGTGAGGTTCACTGTTTCT |
| ***Mus VCAM1*** | AGTTGGGGATTCGGTTGTTCT | CCCCTCATTCCTTACCACCC |
| ***Mus ICAM1*** | GTGATGCTCAGGTATCCATCCA | CACAGTTCTCAAAGCACAGCG |
| ***Mus PECAM1*** | ACGCTGGTGCTCTATGCAAG | TCAGTTGCTGCCCATTCATCA |
| ***Mus NF-κB*** | AGGCTTCTGGGCCTTATGTG | TGCTTCTCTCGCCAGGAATAC |
| ***Mus CCL5*** | GCTGCTTTGCCTACCTCTCC | TCGAGTGACAAACACGACTGC |
| ***Mus Arg1*** | CTCCAAGCCAAAGTCCTTAGAG | AGGAGCTGTCATTAGGGACATC |
| ***Mus eNOS*** | GGCTGGGTTTAGGGCTGTG | CTGAGGGTGTCGTAGGTGATG |
| ***Mus TGFβ*** | CTCCCGTGGCTTCTAGTGC | GCCTTAGTTTGGACAGGATCTG |
| ***Mus IL-4*** | GGTCTCAACCCCCAGCTAGT | GCCGATGATCTCTCTCAAGTGAT |
| ***Mus IL-10*** | GCTCTTACTGACTGGCATGAG | CGCAGCTCTAGGAGCATGTG |
| ***Mus Mrc1*** | CTCTGTTCAGCTATTGGACGC | CGGAATTTCTGGGATTCAGCTTC |
| ***Mus*** ***PPARγ*** | TCGCTGATGCACTGCCTATG | GAGAGGTCCACAGAGCTGATT |
| ***Mus CCL17*** | GACGACAGAAGGGTACGGC | GCATCTGAAGTGACCTCATGGTA |
| ***Mus FOXP3*** | CCCATCCCCAGGAGTCTTG | ACCATGACTAGGGGCACTGTA |
| ***Mus CCL22*** | AGGTCCCTATGGTGCCAATGT | CGGCAGGATTTTGAGGTCCA |
| ***Mus VEGF*** | CTGCCGTCCGATTGAGACC | CCCCTCCTTGTACCACTGTC |
| ***Mus MMP7*** | CTGCCACTGTCCCAGGAAG | GGGAGAGTTTTCCAGTCATGG |
| ***Mus MMP12*** | GAGTCCAGCCACCAACATTAC | GCGAAGTGGGTCAAAGACAG |
| ***Mus MERTK*** | ACCCAGTTGCTAGAGAGCTG | TGGTGAGTCTGTCTCCGGTAA |
| ***Mus AMPKα*** | GTCAAAGCCGACCCAATGATA | CGTACACGCAAATAATAGGGGTT |
| ***Mus miR-33*** | GGCACTACTTCTGATCCTTC | CAACTACAATGCACCACAGCTG |
| ***Mus HMGCR*** | TGTTCACCGGCAACAACAAGA | CCGCGTTATCGTCAGGATGA |
| ***Mus*** ***LXRα*** | CTCAATGCCTGATGTTTCTCCT | TCCAACCCTATCCCTAAAGCAA |
| ***Mus LXRβ*** | ATGTCTTCCCCCACAAGTTCT | GACCACGATGTAGGCAGAGC |

IL-1β: interleukin-1β ; TNFα: tumor necrosis factor α; IL-6: interleukin-6; iNOS: inducible nitric oxide synthase; GAPDH: glyceraldehyde-3-phosphate dehydrogenase; IFNγ: interferon gamma; COX2: prostaglandin-endoperoxide synthase 2; TLR4: toll like receptor 4; MCP-1: monocyte chemotactic protein 1; NLRP3: NLR family pyrin domain containing 3; IL-8: Interleukin-8; IL-12: Interleukin-12; VCAM1: vascular cell adhesion molecule 1; ICAM1：intercellular adhesion molecule 1; PECAM1: platelet and endothelial cell adhesion molecule 1; NF-kB: nuclear factor kappa B; CCL5: C-C motif chemokine ligand 5; ARG1: arginase 1; eNOS: endothelia NO synthase; TGFβ: transforming growth factor, beta 1; IL-4: interleukin-4 ; IL10: interleukin 10; Mrc1: mannose receptor C-type 1; PPARγ: peroxisome proliferator activated receptor gamma; Chi3I3: chitinase-like 3; CCL17: C-C motif chemokine ligand 17; FOXP3: forkhead box P3; CCL22: C-C motif chemokine ligand 22; VEGF: vascular endothelial growth factor; MMP7: matrix metallopeptidase 7; MMP12: matrix metallopeptidase 12; MERTK: MER proto-oncogene, tyrosine kinase; HMGCR: 3-hydroxy-3-methylglutaryl-CoA reductase; LXRα: Liver X receptor α; LXRβ: Liver X receptor β.
